# Case of Abortion

**Published:** 1859-12

**Authors:** W. Johnson

**Affiliations:** White House, N. J.


ambition to strive thus to survive on earth the brief term of mortal life!

The career of the distinguished man whose biography has been our theme on this occasion, is pre-eminently worthy of admiration. In his character were beautifully blended the finest intellectual and moral qualities of our nature. With mental powers of the highest order, were combined simplicity, modesty, purity and disinterestedness, in such measure that we feel he was a man to be loved not less than admired. His zeal and industry in scientific pursuits were based on the love of truth for its own sake, and a desire to be useful to his fellow men. To these motives to exertion much of his success is to be attributed. Mere intellectual ability and acquirements do not qualify either to make or to appreciate important scientific discoveries. The mind must rise above the obstructions of self-love, jealousy and selfish aims. Hence it is, that most of those who have attained to true eminence in the various paths of scientic research, have been distinguished for excellencies of the heart as well as of the head.

The example of Laennec is worthy of our imitation. His superior natural gifts we can only admire. His mission was far above aught that we can hope for. But we can imitate the industry, without which, his genius would have been fruitless. We may aspire to emulate his virtues. It is neither arrogance nor folly to be inspired by ambition in contemplating intellectual and moral attainments which are beyond our reach. We are commanded ever to strive for Divine perfection. Let us show our reverence for the memory of Laennec by endeavoring to follow humbly in his footsteps.—*New Orleans Medical News and Hospital Gazette.*

---

*Case of Abortion.* By Dr. W. Johnson, of White House, N. J.

Dec. 3, 1859, I was called in haste about 5 o'clock last evening to the wife of W. V. She is about 30 years of age, and the mother of four children. I found her flooding most profusely. Upou inquiry, she informed me that she was enciente about three months. I immediately requested an examination per vaginam. As I was about introducing my finger for this purpose, a prodigious discharge of blood took place; as if the vis a tergo of fluid blood had broken through the coagulum which was damming up the vagina. I am accustomed to encountering large discharges of blood, but this was most appalling. I passed my finger however on to the os uteri. Here I found the produce of conception engaged; but I could not disengage and bring it away without endangering its being ruptured. I preferred letting it alone for the present, and trusting awhile to the tampon, in the belief that the contractions of the uterus would bring the mass into the vagina, from whence it could very readily be removed. I tore up three or four long strips of old muslin of the width of my hand, and passing up one after another with my fingers I completely filled up the vagina. The hemorrhage was now entirely restrained. The patient had fainted, but had revived. I had cloths dipped in cold vinegar and applied over the abdomen, and dirrected them to be removed as soon as they became warm, and re-applied cold. I gave her 15 grs. of pulverized ergot with 3 grs. of plumbi acetas, and directed half this quantity of the ergot, and the three grs. of p. acet. to be given every half hour, until she was again seen. My son, Dr. J. V. Johnson, saw her again in the evening, and left her at 9 o'clock doing well. He directed the continuance of the medicines a couple of hours longer.

I saw the patient again early in the morning, and found her doing well. Perhaps not more than an ounce of blood had been discharged. She had been kept cool, very quiet and restrained from motion and conversation. She said that she felt well; her pulse was normal. She informed me, however, that she had had considerable pain soon after my son left, doubtless from the action of the ergot. I now removed the tampon, and found what I expected; the whole product of conception in the vagina.—With my two fingers, I very readily removed it. It was a beautiful specimen of early fœtation; it was of the size of a large hen's egg. The mass was entire. The chorion was as thick as a knife-blade; the amnion was very thin, and its liquor very transparent. The fœtus was of the size of a bumble bee.

I directed the patient to keep her bed a few days, and take on the following day a small dose of ol. ricini.

*Remarks.*—I have related this case not so much for its novelty as for its practical importance. It strikingly illustrates the great valure of the tampon as an obstetrical appliance. Had it not been promptly resorted to in the case just related, I honestly believe that death would have resulted from the omission.—The tampon rescued her from a state of imminent danger and placed her in one of complete security. I shall never forget the deep solicitude which I experienced at my first case of ante partum hemorrhage. I was then a very young man; the life of my patient and my own reputation were at stake. About forty-seven years ago I was called to Mrs. B. V. H. in a miscarriage in her fifth month. This misfortune had happened to her as often as a dozen times at different periods of her gestation. I found her flooding very much, and with very little pain. From the high commendation bestowed on the tampon by distinguished accoucheurs, I was induced to resort to its use. The pains increased in force, and by and by became bearing down. I now withdrew the tampon, and very rapidly two children and the placenta were expelled. The patient did well. From that time to this, I have resorted to the tampon in all appropriate cases.—By this term, I mean *profuse hemorrhage in the early months of utero-gestation.* I do not however consider the tampon to be necessary in every case of abortion—far from it. The hemorrhage may be so slight as not to need interference with it; nature in the majority of cases is sufficient for her own work. We may often assist her by removing with the finger one of the most frequent sources of hemorrhage at this time—namely, the placenta arrested in the os uteri. Dewees' hook may also be used for the same purpose. I have myself succeeded to admiration with this appliance, but I have sometimes been sadly disappointed with it; and I may say the same thing of the uterine forceps.

The tampon possesses one benefit which should not be overlooked. It is this. The necessity of a tedious attendance upon the patient is done away with by its employment. If the vagina be well stuffed, the accoucheur may leave his patient to attend to other duties. In my practice I leave the tampon in the vagina from twelve to twenty hours. There is little or no danger to be apprehended in these cases from concealed hemorrhage.—Candor however obliges me to say that others have come to a different conclusion on this point. I give my own experience, and I have never seen unpleasant consequences from leaving the tampon in the vagina from twelve to twenty hours. I acknowledge that if this resource of our art be used in advanced gestation, the patient should never be left by her attendant; nor an inspection of her abdomen be neglected; she may fall a victim to concealed hemorrhage.

As to the modus operandi of the tampon, I think it is something more than a simple plug arressting the flow of blood and favoring its coagulation. By the irritation of its presence in the vagina, the uterus is sympathetically awakened up to action.

With respect to the material for making tampon, I have not myself been very particular. I have used for this purpose a silk handkerchief; pieces of old muslin either torn in strips or used whole. I however give the preference to muslin torn up in strips. I have generally greased them before their introduction.

[*Philadelphia Medical & Surgical Reporter.*

